# An asymptomatic mutation complicating severe chemotherapy-induced peripheral neuropathy (CIPN): a case for personalised medicine and a zebrafish model of CIPN

**DOI:** 10.1038/npjgenmed.2016.16

**Published:** 2016-06-08

**Authors:** Michael P Holloway, Bradley D DeNardo, Chanika Phornphutkul, Kevin Nguyen, Colby Davis, Cynthia Jackson, Holly Richendrfer, Robbert Creton, Rachel A Altura

**Affiliations:** 1 Department of Pediatrics, Division of Pediatric Hematology-Oncology, Hasbro Children’s Hospital and The Warren Alpert Medical School at Brown University, Providence, RI, USA; 2 Department of Pediatrics, Division of Pediatric Endocrinology and Metabolism, Rhode Island Hospital and Brown University, Providence, RI, USA; 3 Departments of Pathology and Clinical Molecular Biology, Rhode Island Hospital and Brown University School of Medicine, Providence, RI, USA; 4 Department of Molecular Biology, Cell Biology and Biochemistry, Brown University, Providence, RI, USA

## Abstract

Targeted next-generation sequencing (NGS) identified a novel loss of function mutation in *GARS*, a gene linked to Charcot–Marie–Tooth disease (CMT), in a paediatric acute lymphoblastic leukaemia patient with severe chemotherapy-induced peripheral neuropathy (CIPN) due to vincristine. The patient was clinically asymptomatic, and lacked a family history of neuropathy. The effect of the mutation was modelled in a zebrafish knockdown system that recapitulated the symptoms of the patient both prior to and after treatment with vincristine. Confocal microscopy of pre- and post-synaptic markers revealed that the GARS knockdown results in changes to peripheral motor neurons, acetylcholine receptors and their co-localisation in neuromuscular junctions (NMJs), whereas a sensitive and reproducible stimulus–response assay demonstrated that the changes correlating with the GARS mutation in themselves fail to produce peripheral neuropathy symptoms. However, with vincristine treatment the GARS knockdown exacerbates decreased stimulus response and NMJ lesions. We propose that there is substantial benefit in the use of a targeted NGS screen of cancer patients who are to be treated with microtubule targeting agents for deleterious mutations in CMT linked genes, and for the screening in zebrafish of reagents that might inhibit CIPN.

## Introduction

The vinca alkaloid vincristine is an essential component of the treatment regimen for a range of malignancies including acute lymphoblastic leukaemia, lymphoma and several childhood solid tumours.^1^ It is a microtubule elongation inhibitor that acts by binding to β-tubulin and preventing microtubule polymerisation.^2^ Unlike other chemotherapy medications, most of which have a dose-limiting toxicity of myelosuppression, the dose-limiting toxicity of microtubule targeting agents (MTA) including vinca alkaloids and taxanes is chemotherapy-induced peripheral neuropathy (CIPN). Symptoms can range from sensory neuropathy, paraesthesia, diminished or absent reflexes, and constipation to chronic severe pain and weakness in distal limbs.^3,4^ Patients with a hereditary neuropathy treated with vincristine can develop exaggerated and long lasting, or irreversible, CIPN symptoms including paralysis.^5^ However, most paediatric cancer patients presenting with severe, even fatal, CIPN do not have symptoms, or a family history, of neuropathy.^6,7^ The vulnerability of the long motor neuron axon microtubules to disruption by MTAs has been central to proposed mechanisms for CIPN. Vincristine has been shown to inhibit fast axonal transport along those microtubules.^8^ Others have reported that disruption of microtubules by prolonged exposure to vinca alkaloids causes Wallerian degeneration, or ‘dying back’, of motor axons similar to that observed in axon crush injuries, producing a decrease in innervation.^9^

Charcot–Marie–Tooth (CMT) is the most common hereditary peripheral neuropathy with an estimated prevalence of 1 in 1,200 to 1 in 2,500.^10^ In a 2003 Children’s Oncology Group study 10% of patients with severe vincristine induced neuropathy were discovered to have had a family history, clinical findings, or genetic evidence of CMT.^11^ Although the authors concluded that the presence of hereditary peripheral neuropathy should be determined in order to individualise the treatment, these studies have not been performed and no formal recommendations for clinical or genetic testing prior to initiating anti-cancer therapy have yet been made. The true prevalence of asymptomatic mutations linked to CMT is presently unknown but likely underestimated. Despite reported cases of severe vincristine-induced neuropathy in patients with CMT following only one or two doses of medication, or a dose <2 mg,^12,13^ the risk of neurotoxicity within this group remains underappreciated.

CMT has been classified into several subtypes based on pathophysiology. One such subtype, CMT2D, is an autosomal dominant disease linked to mutations within the glycyl-tRNA synthetase (*GARS*) gene. GARS is an aminoacyl-tRNA synthetase protein that catalyses the addition of glycine to transfer RNA (tRNA). Motor neurons are affected in all cases. Affected patients generally have normal sensory, and normal to mildly slowed motor nerve conduction velocities. Biopsies show an axonal pathology.^14,15^ Defining the pathologic mechanism is complicated by the ubiquitous expression of GARS, a lack of a known axon specific function for GARS, and the lack of correlation between an aminoacylation defect and the linked phenotype.^16^ For the interpretation of our observations here, it is important to note that all *GARS* mutations linked to CMT2D are dominant and that a mouse heterozygous transgenic line engineered with an ~50% reduction in GARS expression had no CMT phenotype.^17,18^ This makes a loss of function pathogenesis for GARS-linked CMT unlikely and a dominant toxic, gain of function, effect of the mutation a more likely cause of CMT2D. As with the mouse model of haploinsufficiency, the patient described here does not present with CMT.

*Danio rerio* is an established model organism for the study of neuroactive small molecules,^19^ neural development^20^ and neurodegenerative disease.^21^ The exome sequence is highly conserved between zebrafish and human, and the GARS messenger RNA (mRNA) is 76% similar. Zebrafish stimulus–response assays have previously been proposed as a rapid and inexpensive vertebrate model system for pharmaceutical screening.^22^ Khan *et al.*, recently used zebrafish to show axon growth and behaviour abnormalities after exposure to vincristine.^23^ Of particular interest to us are the reports using zebrafish to study development of, and injuries to, neuromuscular junctions (NMJs).^24,25^ Prior work from our group and others showed that high-throughput assays can be used to test the acute effects of neurotoxic agents by analysing behaviour responses of zebrafish larvae.^26,27^ With this paper we hope to show that a zebrafish model, using the methods displayed here, could be a relatively rapid, cost effective and highly informative tool for investigating and treating CIPN and predisposing complications.

Here we describe a method for quickly evaluating a clinically discovered unique gene variant suspected of predisposing a patient to severe CIPN. A novel heterozygous gene mutation in *GARS* was identified by a diagnostic test in a child with leukaemia who developed a Grade IV vincristine-induced neuropathy. Analysis of patient lymphocytes and patient-derived lymphoblasts demonstrate that this mutation results in the formation of an unstable protein product resulting in significantly reduced levels of the protein. We demonstrate the use of a zebrafish model system to evaluate the functional significance of this one variant. Morpholinos were used to knockdown GARS protein expression and the effects on zebrafish motor function, axon development, and NMJs were examined. Similar to the patient, the GARS knockdown fish displayed no CIPN-like symptoms, but combined treatment with vincristine produced a neuropathic phenotype.

## Results

A 12-year-old female of Swedish-Eastern European descent with no significant past medical history presented in septic shock with multisystem organ failure secondary to *Escherichia coli* and *Bacillus cereus* bacterium. She was pancytopenic with blast forms on her peripheral blood smear. Bone marrow aspirate confirmed the diagnosis of pre-B-cell acute lymphoblastic leukaemia. At presentation, she had no neurologic complaints and family history was only significant for a maternal grandmother with multiple sclerosis. She began standard induction chemotherapy with vincristine, doxorubicin, methotrexate, asparaginase and prednisone. Following the second dose of vincristine (cumulative dose 4 mg) she reported severe back pain and constipation. She experienced a gradual worsening of peripheral neuropathy, including generalised muscle weakness, foot drop and frequent falling. She developed paraesthesias of both hands, atrophy of her thenar muscles and inability to hold objects or ambulate without assistance. She subsequently became wheel-chair bound. Vincristine was held after a cumulative dose of 12 mg. She was readmitted for further evaluation. Physical examination revealed significant muscle weakness, steppage gait and loss of DTRs. Muscle weakness was predominantly distal, although proximal weakness was graded 4/5 bilaterally. Sensory exam and cranial nerves were intact.

**Figure 1:**
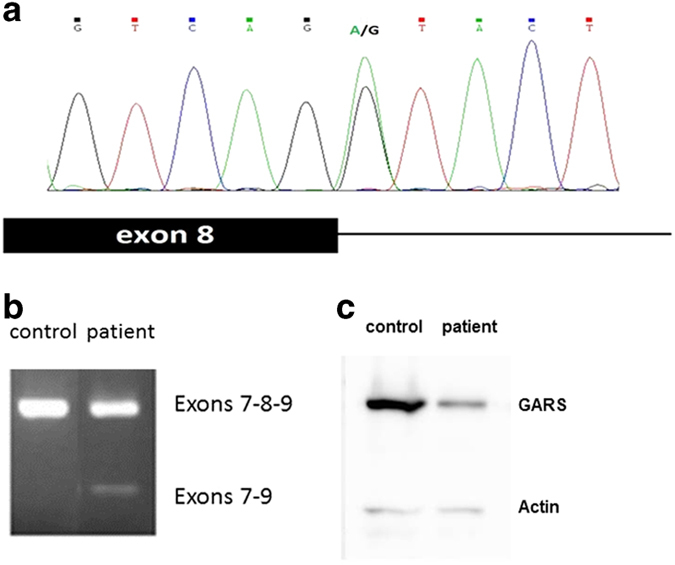
Results of targeted sequencing screen within a set of genes causing hereditary peripheral neuropathy. (**a**) Sanger sequencing revealed a transition point mutation within the exon 8 splice donor site in one copy of the patient’s *GARS* gene. The diagram above shows the sequence trace and the single allele point mutation relative to the position of the 3ʹ end of exon 8. The diagram below illustrates the relative position of this point mutation. (**b**) PCR products using DNA isolated from patient and control lymphocytes and primers in the 5ʹ of exon 7 and the 3ʹ end of exon 9. The product sizes correspond to the expected sizes of spliced exons 7, 8 and 9, and alternatively spliced exons 7 and 9. (**c**) Western blot of total protein extracted from patient and control lymphocytes and probed with antibodies to GARS and control β actin. Approximate densitometry values for GARS relative to actin: control=12.3, patient=4.3.

Owing to the severity of the CIPN, and uncertainty of how best to continue treatment, an evaluation to determine the cause of this child’s peripheral neuropathy was undertaken. Targeted exome NGS of 15 CMT-associated genes revealed a novel heterozygous sequence variant in the *GARS* gene (IVS8+1 G>A mutation) that we validated by Sanger DNA sequencing (Figure 1a). This sequence variant has not previously been reported in either dbSNP or the ExAC human exome sequence database. The remainder of the exome analysis failed to reveal any variant that was likely to be pathogenic. PMP22 and Cx32 duplication/deletion was absent. DNA from both parents were sequenced, revealing an identical mutation in the patient’s father. Nerve conduction studies in the father revealed borderline slowing of motor nerve conduction velocities in the peroneal and tibial nerves. This diagnostic test could not be conducted on the patient, as the patient was already presented with neuropathy, and nerve conduction studies are not generally performed on paediatric patients due to the pain and discomfort involved.

The sequence variant we identified in the *GARS* gene creates a mutation within the 3ʹ donor splice site of exon 8 (Figure 1a). To perform functional studies, we created an immortalised lymphoblastoid cell line from the patient for complementary DNA (cDNA) synthesis. PCR across exon 7 to exon 9 showed an alternate transcript that was not present in the control samples (Figure 1b). Sequencing of this PCR product showed a novel splice product joining exon 7 in frame with exon 9. Further investigation of alternative splicing from putative cryptic splice sites, or two small putative alternative exons that are reported once each in the UCSC Human Genome browser EST tracks, failed to discover any products.

A predicted protein product from the mutant allele transcript would be 5.6 kDa smaller than the wild type. Western blot analysis performed with patient and control lymphoblastoid cell homogenate and probed with a monoclonal antibody to a conserved N-terminal domain of GARS, detected only one band corresponding to wild-type protein (Figure 1c), but with a significant reduction in protein abundance in the patient sample compared with total protein loaded as well as to relative abundance of β-actin. On the basis of these findings, we concluded that the result of the splice donor site mutation is a haploinsufficiency of GARS protein.

A hypothesis based on the known function of GARS in protein synthesis, is that a mutation in this acyl-tRNA-transferase likely causes a defect in translational machinery that could manifest as a disruption in the cell’s metabolic and/or growth function. We attempted to examine this hypothesis by analysing the growth rates of the patient-derived lymphoblastoid cell line versus control. Results of these studies showed no differences between the growth rates of the two cell types, suggesting a lack of generalised deficiency due to *GARS* haploinsufficiency (data not shown).

**Figure 2:**
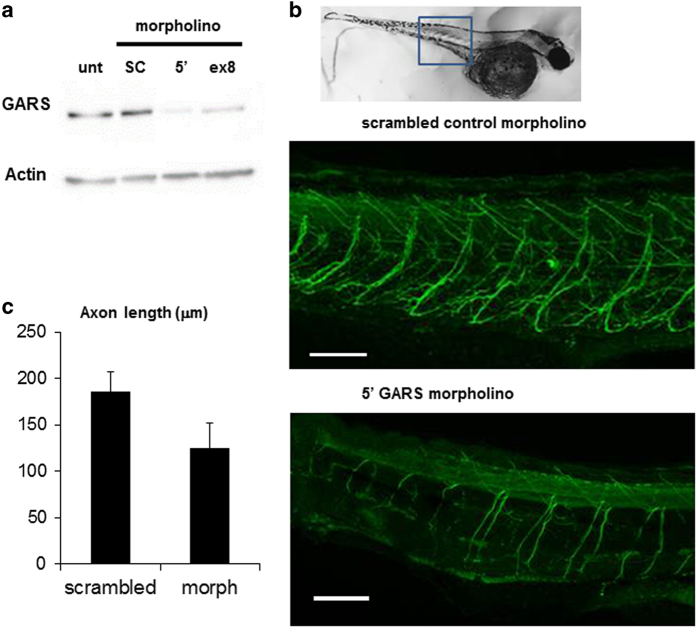
Effect of GARS targeting morpholinos on the development of zebrafish motor axons. (**a**) Western blot of protein extracted from 48 h.p.f. larvae probed with anti-GARS and loading control anti- β actin antibodies. unt=untreated, SC=scrambled morpholino, 5ʹ=GARS morpholino targeting the 5ʹ untranslated sequence, ex8=GARS morpholino targeting the splice donor site of GARS exon 8. Approximate densitometry values for GARS relative to actin: unt=1.1, SC=1.4, 5ʹ=0.1, ex8=0.2. (**b**). Confocal stack z projections of 48 h.p.f. larvae that had been injected with morpholino at the one to four cell stage embryo and stained for acetylated α-tubulin. Above is a brightfield image of a 48 h.p.f. untreated larvae. The box indicates the area on the larvae used for imaging. (**c**). CaP motor axon lengths were measured in the tail just posterior to the egg sac where the dorsal to ventral width of the tail is approximately constant. Four axons were measured in 5 larvae that had been injected with scrambled control or 5ʹ untranslated targeting morpholino. Scale bars are approximately 50 μm. *T*-test *P*=8.48181E-11.

In an effort to study the putative link between the *GARS* mutation and CIPN we used a morpholino (MO) to model GARS haploinsufficiency in zebrafish. We designed GARS MOs to target both the 5ʹ untranslated region of the GARS transcript as well as the exon 8 splice donor site. Both MOs succeeded in reducing the abundance of GARS protein in whole larvae (Figure 2a), mimicking the result of haploinsufficiency in the patient.

To assess the effect of GARS knockdown on motor axon development, we used newly hatched zebrafish larvae (48 h.p.f.) stained with axon specific anti-acetylated α-tubulin. The results demonstrated a delay in motor axon development in GARS MO injected fish (Figure 2b,c), with no difference seen between untreated and scrambled control injected larvae. No gross anatomical differences were detected in length, dorso-ventral width, and eye size between control and GARS MO (data not shown). This suggests that despite the essentially ubiquitous expression pattern of GARS, axon development appears to be uniquely sensitive to a decrease in copy number.

To examine the potential effects of GARS knockdown on muscle innervation, we used synaptotagmin-2 (SYT2), a synaptic vesicle membrane protein that functions as a calcium sensor in exocytosis,^28^ and muscle acetylcholine receptor (AChR), as markers for the presynaptic and postsynaptic sides of neuromuscular junctions respectively. Co-localisation of these two markers is commonly used to indicate the presence of NMJs.^29,30^

Most studies of zebrafish motor axons focus on the developmental stages around 48 h.p.f. where axon and arborisation growth can be easily followed and quantified. At 72 h.p.f., the main trunks of the peripheral motor axons have been established, the hemisegments, myotome and myoseptum are clearly visible, and axon branches have spread throughout the lateral muscle mass. (Supplementary Figure S1). Although the larvae will still be growing and adding muscle mass till reaching maturity, the initial development of the neuromuscular axons and synapse formation is complete, and larvae exhibit mature motor behaviour, after 72 to 96 h.p.f.^31^

**Figure 3:**
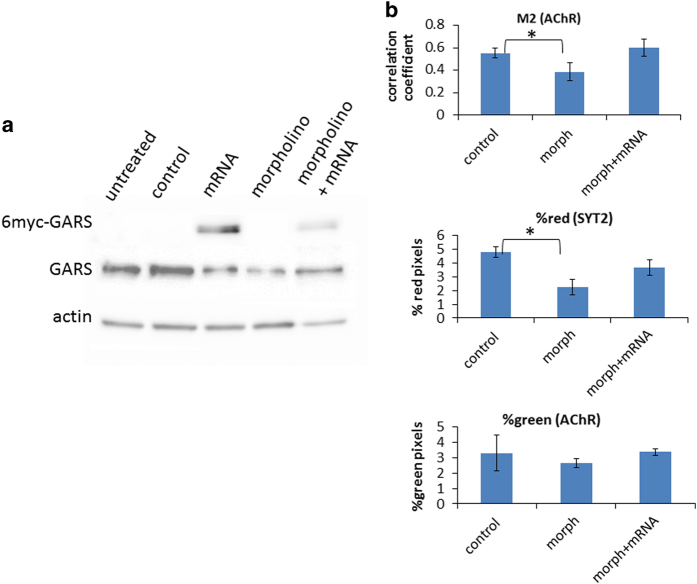
Restoration of SYT2 expression and receptor colocalisation in GARS knockdown fish by co-injection of GARS mRNA. (**a**) 48-h.p.f. larvae were homogenised and equal amounts of protein loaded on a PAGE gel for western blotting. 6Myc=6 copies of the c-myc tag. Approximate densitometry values for GARS relative to actin: untreated=2.4, scrambled control=2.2, mRNA injected=1.3, morph=0.8, morph+mRNA=2.3. Values of ectopically expressed GARS relative to actin: mRNA=1.9, morph+mRNA=0.8. (**b**) 72-h.p.f. larvae were stained with SYT2 antibody and Cy3-conjugated α-bungarotoxin. Z projections of five optical slices in the lateral muscle were made and the myoseptum cropped out. 4 to 6 images for each treatment were used for particle and colocalisation analysis. Error bars represent s.e. GARS knockdown decreases SYT2 expression and acetylcholine receptor colocalisation. Co-injection of GARS morpholino and GARS mRNA-restored colocalisation of α-bungarotoxin with SYT2 and increased expression of SYT2. Red=SYT2. Green=α-bungarotoxin. Asterisk indicates two-tailed *t-*test *P*<0.05. M2=Manders M2 correlation coefficient here measuring the degree of colocalisation of AChR.

Particle analysis at 72 h.p.f. of SYT2 and AChR staining within ~1-μm confocal slices extending into the lateral muscle of the myotome, along with colocalisation analysis of the two stains, revealed that the GARS knockdown significantly reduces the extent of axon outgrowth into the lateral muscle while the concentration of AChR remains essentially unchanged (Figure 3b). On average, this produces a small but statistically significant decrease in co-localisation that is indicative of a decrease in innervation. Initial attempts to quantify axon length and arborisation showed no difference between controls and treated. Only a restricted region of interest within a relatively small portion of the muscle mass provided useful data. No difference between MO treatment and control could be found along the myoseptum, or along the central axon trunk and branches proximal to the spinal cord (data not shown).

To assess the contribution of off target effects to the observations of NMJs after GARS knockdown, we performed rescue experiments by co-injecting wild-type zebrafish GARS mRNA. *In vitro* transcribed GARS mRNA lacking the native 5ʹ untranslated target sequence of the 5ʹ MO was co-injected with GARS MO. A c-myc epitope repeated six times (6myc) on the N-terminus of the ectopically expressed GARS was used to confirm *in vivo* translation (Figure 3a). Image analysis in the lateral muscle shows that the ectopic increase in GARS protein reversed the effects of the GARS MO knockdown (Figure 3b). Both the expression of SYT2 and the co-localisation of SYT2 and AChR markers are increased to the same levels as that of the control MO. This is a strong indication that the decreases observed are due to specific knockdown of GARS protein.

We used an improved stimulus–response assay to examine this effect from vincristine exposure in the presence of a knockdown in GARS expression. Although most zebrafish stimulus–response studies rely on applying a difficult to normalise physical stimulus, such as attempting to touch the fish with a probe, the visual stimulus–response assay method developed by Creton, *et al.* is consistent, automated (and therefore less prone to bias) and reproducible^32,33^ (see Materials and Methods and Supplementary Figure 2).

**Figure 4:**
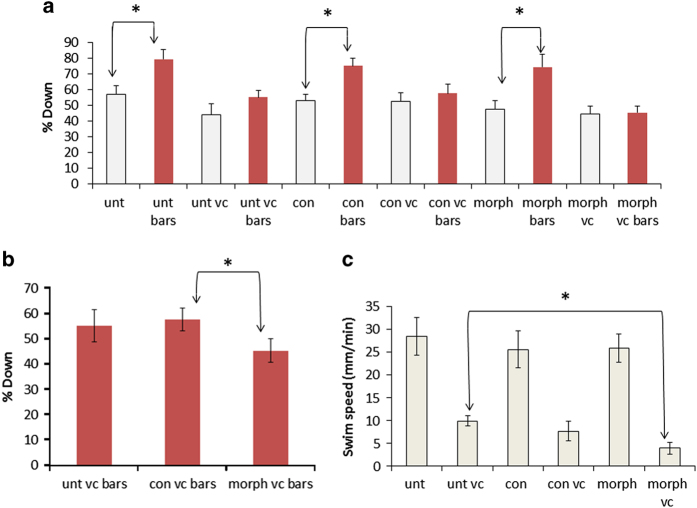
Synergistic effect of GARS knockdown and vincristine on zebrafish stimulus–response. Larvae were exposed to 10 μM vincristine for 24 h prior to assay. At the time of the assay larvae were 96 h.p.f. (**a**) Ratio of larvae grouped in the ‘bottom’ half of the well versus the ‘upper’ half averaged over the observation period. Error bars represent s.e. Asterisks denote *t*-test results where *P*<0.05. Vincristine treatment blocks the ability to respond to the stimulus in both GARS knockdown and controls. (**b**) Some of the same data in the graph above isolated to point out the synergism of the combined knockdown and vincristine treatments. The difference between the control and knockdown is significant with 95% confidence in a one-tailed *t-*test. A one-tailed test is used since the difference being tested is only on one side of the possible distribution. The position of the larva after stimulation is expected to be either unchanged, or larger than 50%. The fish in the combined treatment were less likely to move away from the stimulus. (**c**) In addition to the threefold decrease in speed exhibited by the controls due to vincristine treatment, the combined knockdown and vincristine treated fish have a significant decrease in speed compared with controls. *N*=40–50 larvae for each treatment. unt=untreated, vc=vincristine, con=control, morph=morpholino, untvc=untreated+vincristine.

In the absence of vincristine, GARS MO-treated fish and controls all showed a similar response of avoiding the animated bars (% down, Figure 4a). In the presence of vincristine, however, the GARS knockdown fish displayed significantly lower values for % down than the combination of control MO and vincristine (Figure 4b), suggesting a slowed motor response. A twofold decrease in swim speed was also observed in the GARS MO–vincristine-treated fish compared with control MO and vincristine treatment alone (Figure 4c). These results correlate with the CIPN response of the GARS haploinsufficiency patient and other reports of severe CIPN in patients with CMT-like mutations.

**Figure 5:**
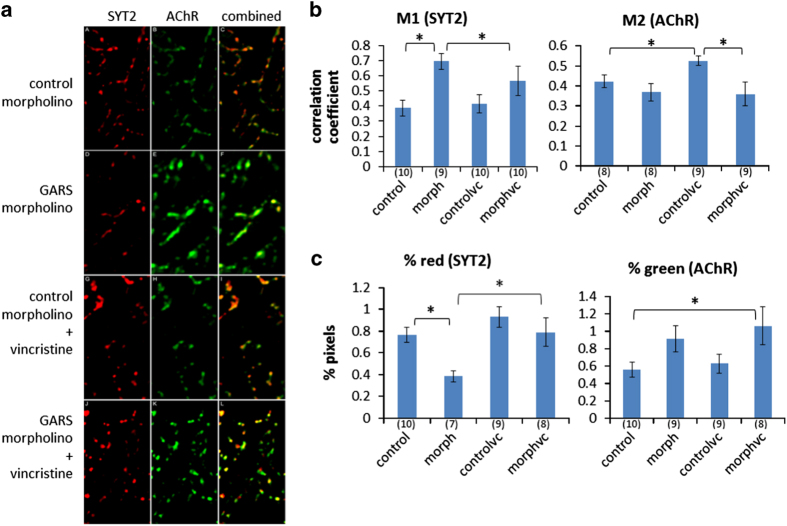
Effects of combined GARS knockdown and vincristine treatments on markers of synapse formation. Larvae were treated with 10 μM vincristine for 24 h prior to fixing at 96 h.p.f. and after the fish had been used for the visual stimulus observations in Figure 4. A Z-projection was prepared for each sample using 10 optical slices from the lateral muscle. The myoseptum was cropped out. 10 images for each treatment were analysed and outliers identified by the modified Thompson tau technique. The number of samples for each average value is given beneath the bars in parenthesis. Error bars are s.e. Two-tailed *t-*test *P* values are as follows: M1 control versus morph *P*=0.001, M1 morph versus morphvc *P*=0.052, M2 control versus controlvc *P*=0.048, M2 controlvc versus morphvc *P*=0.032, % red control versus morph *P*=0.004, % red morph versus morphvc *P*=0.017 and % green control versus morphvc *P*=0.048. Red=SYT2. Green=acetylcholine receptor. morph=morpholino, vc=vincristine. M1=Manders correlation coefficient here measuring the degree of colocalisation of SYT2. M2=Manders correlation coefficient here measuring the degree of colocalisation of AChR.

To correlate the motor effects of the GARS MO in the presence and absence of vincristine with changes in the number of NMJs and peripheral nerves, we conducted confocal microscopy analysis of fluorescently stained motor neurons and post-synaptic markers. The fish used for the behaviour analysis (now at 96 h.p.f.) were fixed and stained and the differences quantified between GARS MO with and without vincristine treatment. The data in Figure 5 are taken from 10 optical slices of the lateral muscle within the myotome extending 10 μm from just beneath the epidermis inward. This is approximately the outer one-tenth of the trunk width in the area examined just posterior of the yolk sac and contains the slow muscle layer and the outer layer of the fast twitch muscle. The myoseptum is cropped out since values along the myoseptum did not change with treatment. Within the Z stack projections axon tips were analysed along with AChR clusters.

As in the previous experiment with 72-h.p.f. larva in Figure 3, in the absence of vincristine GARS knockdown resulted in a twofold decrease in the number of motor axons and an increase in size of the AChR clusters in the lateral muscle (Figure 5 and Supplementary Figure S3). This results in an increase over control of SYT2 co-localising with AChR (Figure 5, M1). The increase in the Pearson’s correlation coefficient indicates an overall increase in co-localisation (Supplementary Figure S3) suggesting an increase in the number, or area, of NMJs. Rather than resulting in a decrease in apparent NMJs, the decrease in axon tips resulting from GARS knockdown seems to be compensated for by the increase in AChR expression and/or clustering resulting in an increase in the NMJ area over that of the control. Therefore, GARS knockdown results in changes to the pre and post-synaptic membranes, but the lack of a resulting decrease in NMJs might be the reason for why there is no measurable effect on the stimulus–response assay (Figure 4). This result correlates with the lack of a neuropathy phenotype due to GARS haploinsufficiency in the patient in the absence of vincristine.

Combining GARS knockdown with vincristine treatment produces a significant increase in axon tips in the lateral muscle mass compared with that seen with the GARS knockdown alone (Figure 5, % red). This change is absent in the control morpholino raised fish when they are treated with vincristine. Rather than producing an increase in co-localisation though, the increase in axon tips is accompanied by a decrease in co-localised pre- and post-synaptic membranes relative to that of GARS knockdown alone (Figure 5, M1). This suggests that under the combined treatment a portion of the axons in the lateral muscle fail to innervate as efficiently as axons in the knockdown fish regardless of their number. This correlates with the reduction in stimulus–response of the GARS knockdown compared with the combined treatment. On the post-synaptic side, despite a significant increase in AChR staining with the combined treatment over that of control, there is no significant change in the amount of AChR co-localised with SYT2 (Figure 5, % green and M2). Similar to the increase in the presynaptic membrane, the AChR increase does not contribute to innervation. It is significant that it is only in the GARS knockdown background that vincristine, at this concentration, has an effect on the markers.

Although there have been proposals of possible treatments for CIPN, there has been little to no data from which to propose clinical trials. For instance, cyclosporine has been proposed due to its inhibition of the mitochondrial permeability transition pore which has been implicated in several neurological diseases.^34^ Calcium-channel blockers FPL64176 and BayK8644 have been used in a zebrafish model of ALS to protect axons from degeneration.^32^

To demonstrate the use of a quantitative zebrafish stimulus–response assay system for screening pharmaceuticals for effects on CIPN, we chose to analyse the effects of the microtubule stabilising drug paclitaxel (brand name: taxol) owing to the opposing effect it has on microtubules compared with vincristine, which is a microtubule-destabilising drug. Neuropathy effects due to paclitaxel have been reported to be absent at a low dose.^33^ We choose a low dose of paclitaxel (10 nM) to approximate an exposure below the effective dose used in chemotherapy. At a similar concentration in cell culture paclitaxel does not increase microtubule mass while nonetheless appearing to stabilise microtubules.^35^

Although 24-h exposure to 10 μM vincristine produced the expected reduction in swim speed and elimination of the stimulus–response, co-administration with low-dose paclitaxel restored both swim speed and the stimulus–response (Supplementary Figure S4), despite having a measurable inhibitory effect when administered alone.

## Discussion

The inherited *GARS* mutation resulting in haploinsufficiency characterised in the patient here could represent either a low-penetrance CMT disease allele, or a small effect size mutation that contributes to a disease phenotype when combined with an additional neuropathic insult, such as that induced by a microtubule-targeting chemotherapy drug. To date, previously described mutations in GARS that are linked to CMT when tested in mouse models result in dominant negative mutant phenotypes. A mouse model of *GARS* haploinsufficiency fails to produce a CMT phenotype.^20^

Although the zebrafish *GARS* knockdown has no obvious disease phenotype, examination of motor neurons and NMJs in the GARS MO-treated zebrafish revealed several differences in development and distribution of pre and post-synaptic markers. First, there is a delay in axon development observed upon hatching. Later, after motor axon development is completed, presynaptic membrane decreases, while AChR clusters increase. Finally, a greater fraction of the presynaptic membrane co-localises with AChR, perhaps caused by the observed increase in AChR. We speculate that this disruption of the normal distribution of NMJs could result in a small decrease in motor nerve conduction without severe neuromuscular dysfunction. However, as decreases in nerve conduction is more closely linked to CMT1 than GARS-associated CMT2,^36^ examination of the myelin sheath in the knockdown would be useful.

The most-promising hypothesis to date of how the disruption of the essential function of GARS could cause a localised lesion in motor axons, involves the observation that wild-type GARS localises along motor axons in granules that are absent when dominant variants are expressed.^37^ Axon functions that depend on local translation along the highly compartmentalised axonal arbours would be partially inhibited in the event of a decrease in local translation and perhaps result in decreased axon length and arborisation. It remains to be determined whether the reduction in expression in our knockdown model would likewise result in a decrease in axon granules containing GARS.

Vincristine treatment resulted in a significant decrease in stimulus response and a change in colocalisation of the NMJ markers. Yet, at these concentrations and length of exposure there is no statistically significant change in either pre- or post-synaptic markers with vincristine treatment alone. Our unexpected result of an increase in apparent NMJs due to vincristine might be explained by a study reporting an increase in co-localisation of pre- and post-synaptic markers in the immediate response of motor axons and postsynaptic membranes to axon damage.^38^ Upon crushing, sprouts form from the motor axons and apparently attempt to reestablish NMJs at AChR clusters. Many of these are not initially functional and therefore would not be able to contribute to a stimulus–response. Therefore, after 24 h of drug exposure it is possible that regeneration following axon damage is occurring that has not yet resulted in fully functional NMJs. A time course examining the effect at intervals shorter than 24 h of exposure could aid in further evaluating this effect.

Although it is currently unclear whether axon regeneration takes place after lesions produced by microtubule-targeting drugs in the same way as it does after physical injury, innervation and NMJs usually recover after chemotherapy with vincristine. In cases of severe CIPN though recovery is limited.^39,40^ Here in our zebrafish knockdown we observe that the pre-existing condition of GARS insufficiency interferes with the motor neuron's response to injury caused by the microtubule-targeted drug. The condition of the NMJs is abnormal prior to administration of vincristine. This could result in less innervation after 24 h of treatment with a relatively low concentration of vincristine. These results suggest that the A>T GARS mutation and subsequent haploinsufficiency could, at least, contribute to the patient’s severe CIPN. Data on the progression of the vincristine-induced lesion, and any regeneration response, immediately after administration of the drug will be essential in understanding the mechanisms involved.

Clinical evidence and technological improvements strongly support routine NGS diagnostics be incorporated into the diagnostic plan and targeted therapy of individual patients in the era of personalised medicine.^41,42^ Our results here suggest that hereditary peripheral neuropathy genes should be added to the targets for sequence analysis among those cancers for which MTA drugs are part of the backbone of cancer therapy. The results of a small trial including 269 patients who were asymptomatic prior to treatment with paclitaxel discovered a benefit to the use of targeted NGS for detecting CIPN predisposition.^43^

Although mammalian disease models have advantages over zebrafish, such as the ability to dose by weight and greater similarity to humans, the zebrafish model system has significant advantages compared with invertebrate and cell culture systems for a rapid, inexpensive experimental system for studying CIPN. If a suspect variant is identified via NGS, then preliminary evidence linking the variant to symptoms can be obtained by modelling the mutation in zebrafish. Both loss and gain of function mutations can be modelled within weeks and evaluated for CIPN symptoms by a sensitive, unbiased and reproducible behaviour assay. Neuromuscular defects can be easily visualised.

As a demonstration of using a quantitative and reproducible zebrafish system to screen drugs that might protect patients from CIPN we have generated results that suggest that paclitaxel at a low concentration, co-administered with vincristine, can inhibit at least some effects of peripheral neuropathy. An ameliorating effect is seen in the stimulus–response assay. We speculate that these effects are due to changes in anterograde fast axonal transport that are known to affect innervation.^44^ A mechanism involving β-tubulin and fast axonal transport is also supported by the pathogenesis of a number of neuropathies and neuronal diseases caused by mutations in β-tubulin that interfere with kinesin binding, as well as mutations within kinesin proteins.^45^ Both drugs bind to distinct sites on β-tubulin. Vincristine’s mechanism of transport inhibition includes the ‘fraying’ dissociation of the plus end of the microtubule.^46^ It’s conceivable that this dissociation could be blocked by paclitaxel stabilising the microtubule strands and thus allowing the last leg of vesicle transport. Alternatively, conformational changes in β-tubulin along the length of the microtubule induced by drug binding might be compensated by both drugs binding to the same, or neighbouring, subunits.

## Materials and methods

### Patient samples and lymphoblastoid cells

Blood samples were obtained after receiving written informed consent from the index patient and family members, and approval by the Internal Review Board of Rhode Island Hospital. Peripheral blood lymphocytes were isolated from the patient. Lymphoblastoid cell lines were derived by The Cleveland Clinic's Genomic Medicine Biorepository.

### Morpholino and mRNA injections

Morpholinos targeting the 5ʹ untranslated region of zebrafish GARS, the exon 8 donor splice site, and scrambled controls were selected and synthesised by Gene Tools (http://www.gene-tools.com). GARS 5′ untranslated: 5′-TGCGCTACACAGAGACAGCATGGAC-3′, GARS 5′ untranslated scrambled control: 5′-TGCcCTAgACAcAGACAcCATcGAC-3′, GARS exon8: 5′-AGTCAGTTGTAATCCACACCTAACA-3′, GARS exon 8 scrambled control: 5′-AGTgAcTTcTAATCgAgACCTAACA-3′. Approximately 1 nl of 6 pg/nl morpholino in 0.05% phenol red (Sigma, St Louis, MO, USA) was injected into embryo yolks at the one to four cell stage.

Full-length *D. rerio* GARS cDNA was obtained from Thermo Bioscience (Pittsburgh, PA, USA), amplified by PCR with *Bam*H1 and *Xho*1 ends, and inserted into a pCDNA6 vector 3′ of a 6myc tag (6 concatenated copies of the c-myc epitope). The pCDNA6-GARS vector was linearised and capped RNA synthesised using the Thermo Scientific TranscriptAid T7 kit (#K0441). Poly(A) tailing was done with the Ambion Poly(A) tail kit (AM1350, Ambion, Waltham, MA, USA). Approximately 1 nl of 250 ng/μl RNA was injected into the embryo yolk.

Glass microinjection needles were made by pulling 1-mm capillary filament (World Precision Instruments, Sarasota, FL, USA) with a Pul-1 Micropipette Puller (World Precision Instruments). Injections were made with a Picoliter Microinjector (Warner Instruments PLI-100A) set to 10 to 20 psi.

### Microscopy

Confocal laser scanning microscopy for Figure 4 was conducted using a Zeiss LSM 710 microscope (Zeiss, Thornwood, NY, USA) with a ×20 Plan-Apochromat objective in the Brown University Leduc Bioimaging Facility. For Figure 5, confocal microscopy was conducted with a Nikon C1si confocal microscope (Nikon, Melville, NY, USA).

Image analysis was conducted with the Fiji/ImageJ software package.^47^ For Figures 3 and 5, selected slices of the stack were used to construct a maximum intensity Z projection. Red and green channels were isolated and each channel filtered with the MaxEntropy threshold algorithm, inverted and then used as a mask with the ImageJ Image Calculator function to filter-out signal below the threshold in the corresponding channels of the Z-projection. These masked red and green channels were then analysed with the Fiji Coloc2 function without the auto-threshold option to obtain the Manders correlation coefficients. The images were then converted to binary and used with the ImageJ Analyze Particles function to obtain the per cent coloured pixels value. The particle analysis produces values for the number of pixels with signal above threshold and is insensitive to intensity of the stain.

For Figure 2c axon length measurements, four axons were measured in each of five larvae for both MO and control groups. For Figure 3b, the control and morpholino+mRNA groups averaged the results from four larvae, while the morpholino group had five. For Figure 5, the number of larvae averaged are noted under the *x* axis in parenthesis. Each experiment was repeated at least twice.

### Behaviour assay

Image capture and analysis of zebrafish larvae undergoing visual stimulation was performed as described by Creton *et al.*^48^ Fifty zebrafish larvae per treatment group were placed in troughs prepared with 0.8% agarose in a rectangular culture dish. Larvae were imaged with the plate resting on top of a laptop screen projecting a white background for 15 min followed by a white background with a red bar moving horizontally under one half of the trough. A macro in ImageJ was used to collect larvae positions and the data processed in an Excel spreadsheet (Zebrafish_macro25k available from authors Richendrfer and Creton.).
