# Supplementary material for: An asymptomatic mutation complicating severe chemotherapy-induced peripheral neuropathy (CIPN): a case for personalised medicine and a zebrafish model of CIPN
Source: NPJ Genom Med. 2016 Jun 8;1:16016–. doi: 10.1038/npjgenmed.2016.16 (PMC5685301; doi:10.1038/npjgenmed.2016.16)
Supplement: Supplementary Information [file npjgenmed201616-s1.doc]

# Supplementary Methods

### Zebrafish line, husbandry, and treatments

This study was approved by the Lifespan (Rhode Island Hospital) Animal Welfare Committee and the Brown University IACUC. Wild-type zebrafish lines were maintained and sacrificed according to standard procedures [Westerfield, M., The Zebrafish Book: A Guide for the Laboratory Use of Zebrafish (Danio rerio) Eugene, OR: University of Oregon Press, 2007.] in the laboratory of Robbert Creton at Brown University. Wild type *Danio rerio* were purchased from Carolina Biological and maintained as an outbred line.

Vincristine sulfate (Hospira) was obtained as a 1mM stock solution and diluted to the desired concentration in egg water for larvae treatment. Paclitaxel (Bristol-Myers Squibb) was obtained as a 7mM solution in castor oil. This was diluted to 10 μM in DMSO and used as a stock for working dilutions. Exposures were conducted for 24 hours at 28.5^o^C in 50 ml of egg water.

### Fixing and staining

All incubations and washes were conducted in a 1.5 ml eppendorf tube on a slowly rocking platform mixer and conducted at room temperature unless otherwise specified. Once the larvae had settled to the bottom of the tube liquid could be completely removed by carefully aspirating with a pipette tip. Larvae were fixed in 4% paraformaldehyde/4% sucrose/0.002% CaCl2. Fixed larvae were stored for up to a week at 4^o^C in the above fixative solution. After washing 3 times for 5 minutes in PBST larvae were perforated by incubating at -70^o^C with prechilled acetone for 10 minutes. The wash step above was repeated and larvae incubated 30 minutes in 1 mg/ml trypsin (Sigma Aldrich) in PBST/1mM EDTA. After 2 brief washes in PBST larvae were incubated in 5 mg/ml hyaluronidase (Sigma Aldrich) in PBST for 60 minutes. Larvae were washed twice briefly in PBST and blocked overnight in 10% sheep serum/2% BSA/PBST. Incubation with anti-SYT2 antibody (znp-1 mouse monoclonal from Zebrafish International Resource Center) was done with a 1 to 300 dilution in 5% sheep serum/2% BSA/PBST for 5 hours. Larvae were washed extensively in PBST 4 times for one hour to overnight. After blocking again as above for one hour, larvae were incubated with sheep anti-mouse F(ab’)2 conjugated to Cy3 (Jackson ImmunoResearch) diluted 1 to 400 in 5% sheep serum/2% BSA/PBST for four hours and protected from direct light. Larvae were again washed extensively as after the primary antibody followed by incubation with 10 μg/ml FITC conjugated α-bungarotoxin (Life Technologies/Molecular Probes F-1176) in 5% sheep serum/2% BSA/PBST for 30 minutes. Larvae were washed 4 times for 15 minutes in PBST and flat mounted with Prolong Gold on a glass slide with a #1.5 coverslip.

For tubulin staining, fixed and digested 48 hpf larvae were stained as above with a 1 to 500 dilution of mouse anti-acetylated α-tubulin (Santa Cruz) and Alexa488 conjugated anti-mouse antibody (Jackson ImmunoResearch). Stained larvae were incubated in Prolong Gold overnight at 4^o^C and mounted in 1% agarose/50% glycerol over #1.5 glass in glass bottom culture dishes (MatTek).

### Western blots

For western blots, lymphoblasts were lysed on ice in RIPA buffer (1% NP40, 0.05% sodium deoxycholate and 0.1% sodium dodecyl sulfate in phosphate buffered saline) and protein concentrations determined by BCA [Smith, P. K., Krohn, R. I., Hermanson, A. K., Gartner, M. D., et al. Measurement of protein using bicinchoninic acid. *Analytical Biochemistry* 1985;150:76-85.]. For zebrafish immunoblots, ten 48 hpf larvae per group were ground under liquid nitrogen with mortar and pestle and then lysed on ice in RIPA. Lysates were separated by SDS-PAGE, transferred to PVDF membrane, and blocked in 5% nonfat dry milk/0.3% Tween in PBS. GARS was detected using a mouse monoclonal anti-GARS antibody targeting the highly conserved N terminus of the protein (Santa Cruz sc-365442), and β actin loading control detected with rabbit anti-actin (Cell Signaling Pan-Actin Antibody #4968). Zebrafish expression of myc tagged GARS after GARS mRNA injection was tested via western blot using mouse monoclonal c-myc antibody (Santa Cruz 9E11). Blots were developed with anti-isotype HRP conjugated secondary antibodies (Santa Cruz) and ECL Prime (GE Healthcare Life Sciences).

### Molecular Genetic Analysis

Next generation sequencing using the CLIA approved diagnostic test for CMT associated genetic variants was conducted by Athena Diagnostics (Marlborough, MA). The genes included in the panel of targets comprise those linked to over 85% of reported CMT1 cases, as well as genes linked to CMT2 and CMT4. These are Cx32, MPZ, PMP22, EGR2, NFL, PRX, GDAP1, LITAF, MFN2, SH3TC2, FIG4, LMNA, RAB7, GARS, HSPB1.
Genomic DNA was extracted from whole blood on the Maxwell 16 Automated nucleic acid purification system using the Blood DNA purification kit (Promega, Madison, WI). Traces were analyzed using Mutation Surveyor DNA variant analysis software (Softgenetics, State College, PA). Sequencing of genomic DNA PCR products was accomplished by direct Sanger Sequencing using a standard BigDye Terminator protocol on an automated 3500 ABI Genetic Analyzer (Applied Biosystems, Foster City, CA). Exon 8 donor splice site primers: forward primer in exon 8: CCAGAAACTGCACAGGGGATT, reverse primer in intron 8: TGCCTGTCATCCTGGAGATTACATT.

For cDNA PCR total RNA was purified using the QIAmp RNA blood mini kit followed by DNase treatment using the RNAse free DNase set (Qiagen, Valencia, CA). Randomly primed first strand cDNA synthesis was performed using the Ipsogen RT kit (Qiagen). During cDNA synthesis, simultaneous control reactions lacking reverse transcriptase were run to rule out DNA contamination. PCR primers: forward primer in exon 7: CAGCAAGAACTTGCGGATCTTT, reverse primer in exon 9: GGGTGGTCTTTCTCACTGGG. All products were run on a 3% agarose gel (2% standard agarose 1% NuSieve agarose). Bands were excised and gel purified using the Qiagen Gel Purification kit (Qiagen). The primers used for cDNA PCR were also used for sequencing. cDNA sequencing was done by Eurofins Genomics ([www.operon.com](http://www.operon.com/)).

### Statistics

Behavior and particle analysis groups were compared for significant differences by Student's T-test. For the particle analysis a two-tailed t-test is used. For the behavior analyses a one-tailed t-test is used since the distribution can only be greater than approximately 50%. Outliers in the particle analysis values used in figure 5 were determined by the modified Thompson's Tau method. Error bars represent the standard error. The sample size of 50 for the behavior analyses is in excess of that needed to detect a difference of 10 with a standard deviation of 20 by a paired t-test. The particle analyses are pilot observations for which we estimated that 5 to 10 observations could be combined and tested for significance.

# Supplementary Figures


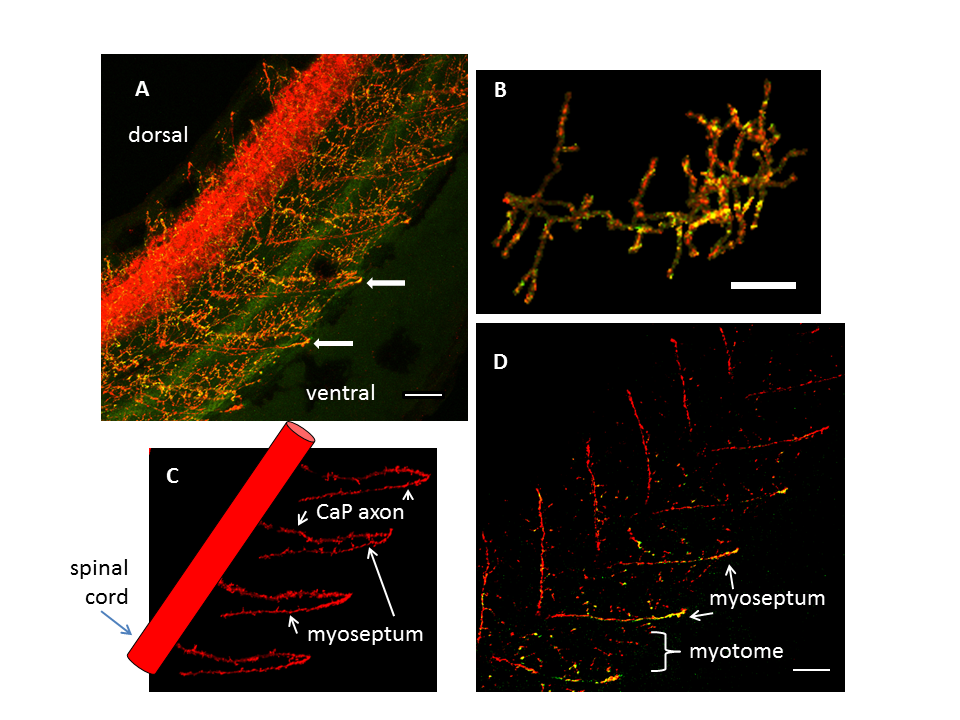


**Supplementary Figure 1:** **Illustration of the area of lateral muscle mass analyzed for synapse quantification. A.** Maximum intensity projection of all slices of the confocal microscope Z stack taken of a 96 hpf untreated larvae. Red fluorescence represents SYT2 primarily expressed in the neurons and green fluorescence represents acetylcholine receptors. Images were made from the tail just posterior of the egg sac. Arrows indicate where the CaP motor neuron loops around the ventral edge of the muscle and then runs along the ventral half of the chevron shaped myoseptum along the lateral edge. **B.** Isolated image of a CaP motor axon and branches starting at the spinal cord and down to the ventral edge traced and filled out over multiple confocal slices using the Simple Neuron Tracer plugin in ImageJ. Points of colocalization can be seen at the tips of branches as well as along the main trunk. **C.** CaP motor axon traced and filled out from its exit from the spinal cord to its end at the horizontal myoseptum. **D.** A single optical slice at the lateral edge illustrating the regions used for image analysis. The tips of the motor axon branches within the muscle can be seen in the myotome region between the bordering myoseptum. Scale bars are approximately 50 μm.

**
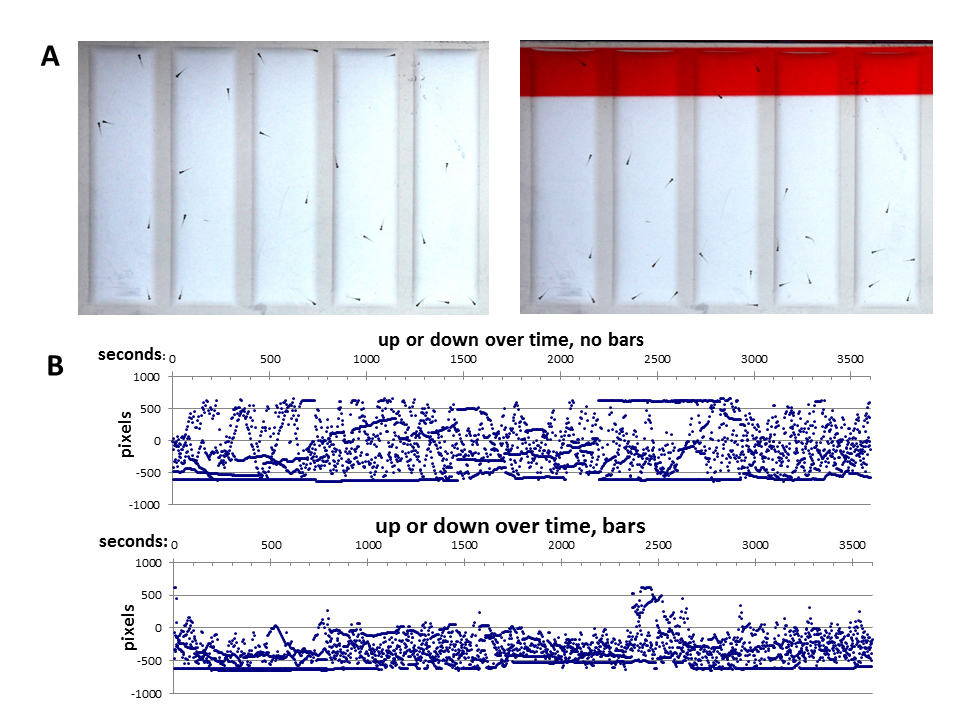
**

**Supplementary Figure 2:** **Illustration of visual stimulus-response behavior assay. A.** Agarose wells cast in a one well plate containing five 96 hpf larvae per well. The plate is illuminated from below by a laptop screen. On the left is one image in a series taken over 30 minutes with no stimulation. After the initial 30 minutes to collect baseline motion data an animated bar is projected that moves across the upper half of the well in an animated reciprocating path for another 30 minutes. **B.** Snapshots from unstimulated (no bars) and stimulated (bars) periods were processed with ImageJ to produce larvae positions relative to the center of the well over time. Comparison of the dot plots of position vs. time of the unstimulated and stimulated untreated control larvae illustrates how the larvae avoid the moving bar and linger in the bottom of the well.

**
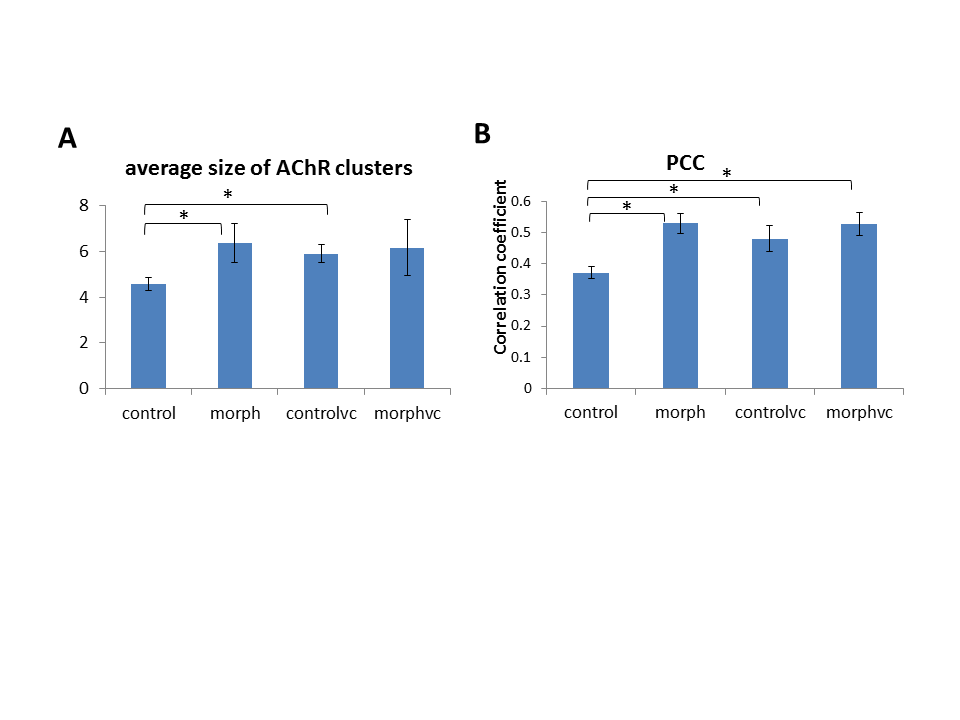
**

**Supplementary Figure 3:** **Effects of combined GARS knockdown and vincristine treatments on AChR cluster size and Pearson’s correlation coefficient**. Larvae were treated with 10 μM vincristine for 24 hours prior to fixing at 96 hpf. A z projection was prepared for each sample using 10 optical slices from the lateral muscle. The myoseptum was cropped out. N = 5. Error bars are standard error. Axis abbreviations: morph = morpholino, vc = vincristine. T test results for PCC values: control vs morph p=0.0006, control vs controlvc p=0.022, control vs morphvc p=0.048.


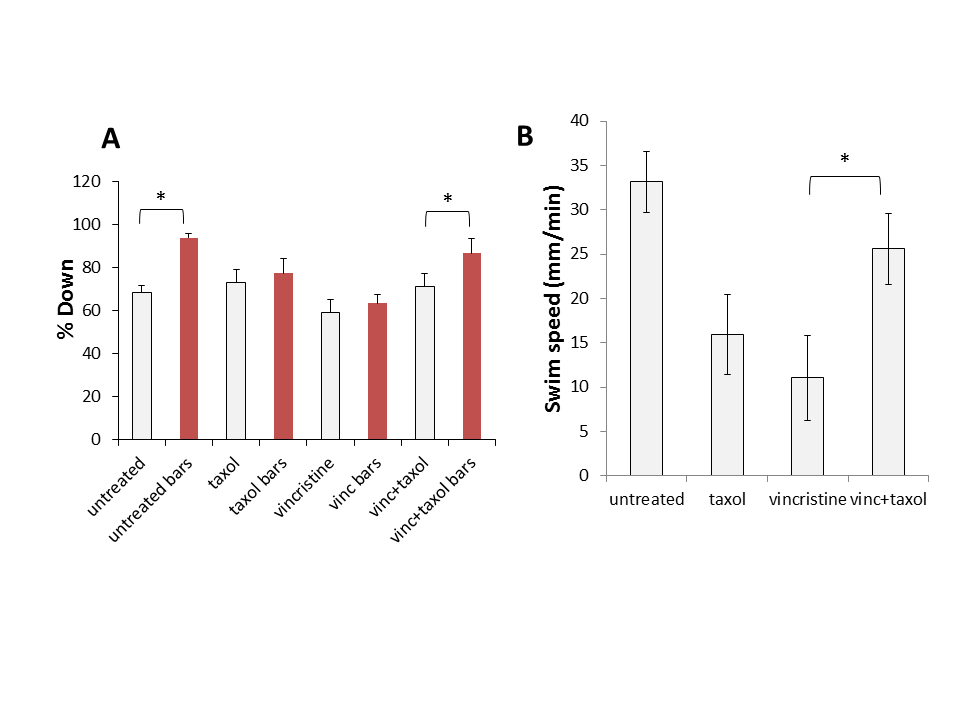


**Supplementary Figure 4:** **Co-administration of paclitaxel (taxol) with vincristine reverses vincristine effects on stimulus-response and swim speed.** Larvae were treated with 10 μM vincristine and/or 10 nM paclitaxel for 24 hours prior to assay. At the time of the assay larvae were 96 hpf. **A.** Stimulus-response results from measurements of the ratio of the larvae location relative to the animated bars moving across the upper half of the well. The combined treatment restores the stimulus response seen in the controls. **B.** The decrease in swim speed seen upon vincristine treatment is significantly reversed with the combined treatment. Axis abbreviations: vinc = vincristine
